# RETRACTION: Treatment of Diabetic Foot Ulcers With External Application of Chinese Herbal Medicine: An Overview of Overlapping Systematic Reviews

**DOI:** 10.1111/iwj.70612

**Published:** 2025-04-21

## Abstract

**RETRACTION:** LiangX. , XiaY. , XuZ. , ZengQ. , GaoG. , HeJ. , XuD. , “Treatment of Diabetic Foot Ulcers With External Application of Chinese Herbal Medicine: An Overview of Overlapping Systematic Reviews,” International Wound Journal 21, no. 4 (2024): e14563, 10.1111/iwj.14563.38135909 PMC10961878

The above article, published online on 22 December 2023, in Wiley Online Library (http://onlinelibrary.wiley.com/), has been retracted by agreement between the journal Editor in Chief, Professor Keith Harding; and John Wiley & Sons Ltd. Following an investigation by the publisher, all parties have concluded that this article was accepted solely on the basis of a compromised peer review process. The editors have therefore decided to retract the article. The authors did not respond to our notice regarding the retraction.

**RETRACTION:** X. Liang , Y. Xia , Z. Xu , Q. Zeng , G. Gao , J. He , D. Xu , “Treatment of Diabetic Foot Ulcers With External Application of Chinese Herbal Medicine: An Overview of Overlapping Systematic Reviews,” International Wound Journal 21, no. 4 (2024): e14563, 10.1111/iwj.14563.38135909 PMC10961878

The above article, published online on 22 December 2023, in Wiley Online Library (http://onlinelibrary.wiley.com/), has been retracted by agreement between the journal Editor in Chief, Professor Keith Harding; and John Wiley & Sons Ltd. Following an investigation by the publisher, all parties have concluded that this article was accepted solely on the basis of a compromised peer review process. The editors have therefore decided to retract the article. The authors did not respond to our notice regarding the retraction.

